# Eosinophilic pustular folliculitis: The successful identification and treatment of clinically and histologically variable presentations

**DOI:** 10.1016/j.jdcr.2026.07.053

**Published:** 2026-08-04

**Authors:** Rafael O. Homer, Madeleine Isler, Keith Morley, Anne M. Stowman

**Affiliations:** a University of Washington School of Medicine, Seattle, Washington; b Division of Dermatology, Department of Medicine, University of Vermont Medical Center, Burlington, Vermont; c Department of Pathology, University of Vermont Medical Center, Burlington, Vermont

**Keywords:** biologic, dupilumab, eosinophil, eosinophilic pustular folliculitis, pediatric, ruxolitinib

## Introduction

Eosinophilic pustular folliculitis (EPF) is a chronic and recurrent inflammatory skin disease distributed primarily on the face, back, and trunk.^1^ On histology, EPF variants are largely indistinguishable and typically demonstrate a predominantly perivascular and perifollicular eosinophilic and lymphocytic inflammatory infiltrate.^2^ There is no standard treatment for EPF, but a variety and combination of treatments such as indomethacin, corticosteroids and antihistamines remain first line and can alleviate both pruritic and cutaneous findings.^3^^,^^4^

We detail a case of EPF in a 2-year-old male with an excellent clinical response to dupilumab and atypical histologic findings, as well as an atypical clinical presentation of dupilumab-resistant EPF with classic histopathology findings in a now 25-year-old female.

## Case presentations

### Case 1

**Fig 1:**
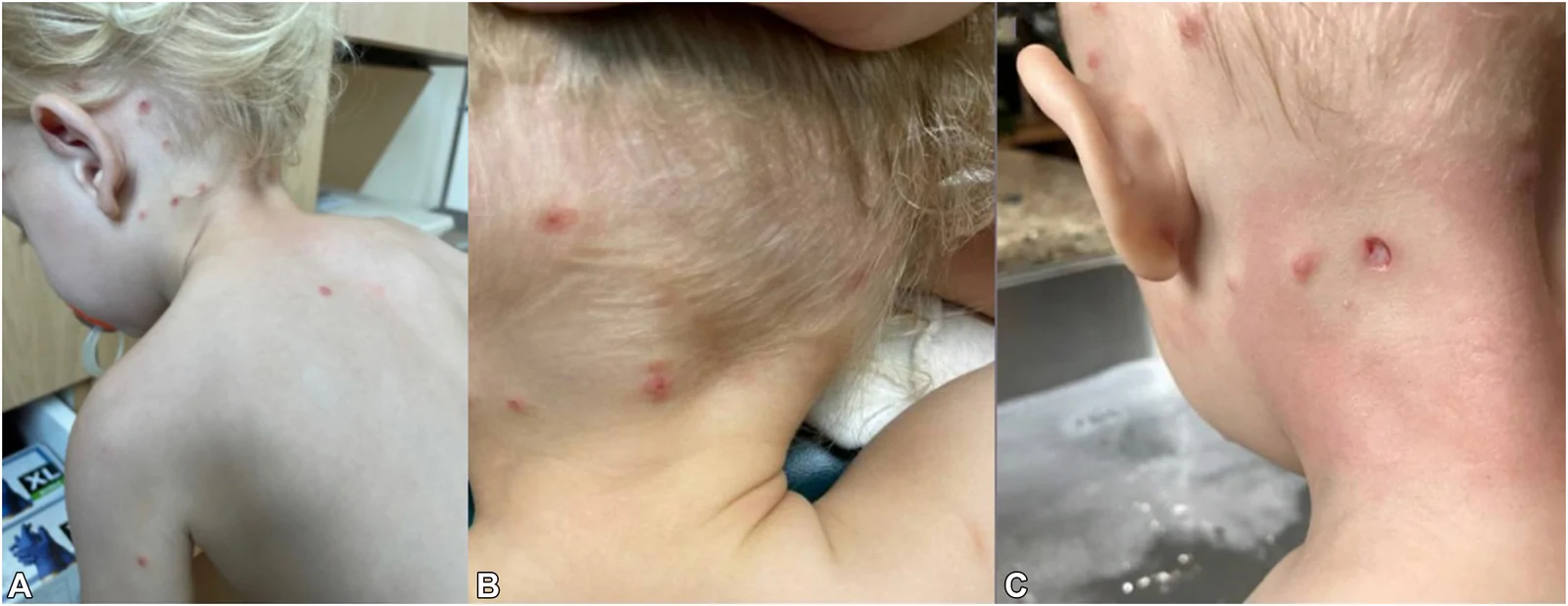
Case 1: Clinical presentation. **A-C,** Scattered, discrete, erythematous-to-brown 3-4 mm papules present at the posterior scalp, upper neck, hairline, posterior ears, upper arms, and back. **C,** Angular excoriated erosions present on scalp and neck.

A 2-year-old male with history of hypoxic ischemic encephalopathy at birth and gastroesophageal reflux disease, presented for dermatologic evaluation of a persistent rash and intermittent fevers lasting 2-3 days for around 2-3 months. Full body clinical examination revealed scattered, discrete, erythematous-to-brown 3-4 mm papules present at the posterior scalp, upper neck, hairline, posterior ears, upper arms, and back (Fig 1). Some lesions had central erosion and serosanguinous crust. The clinical differential diagnosis was broad and included Langerhans cell histiocytosis, lymphomatoid papulosis, pityriasis lichenoides et varioliformis acuta and arthropod bites.

**Fig 2:**
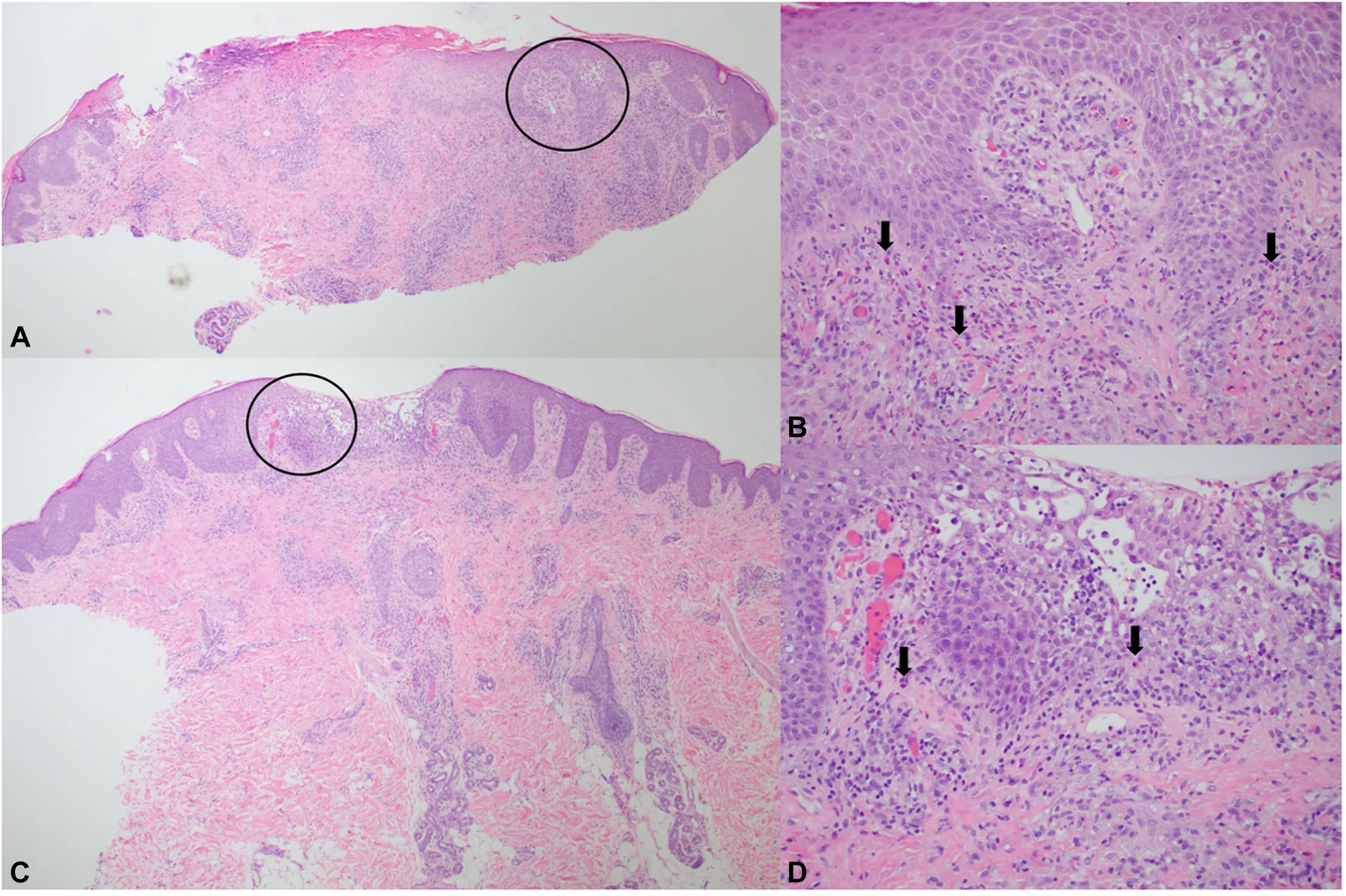
Case 1: Histology. The biopsy of the back lesion **(A)** (4×, H&E) on low-power shows a partially eroded epidermis with areas of spongiosis and overlying parakeratosis and serum crust. Within the dermis, perivascular inflammation is seen, extending into the mid dermis. **B,** (20×, H&E) Higher-power highlights epidermal spongiosis and brisk inflammation containing increased numbers of eosinophils. The biopsy of the arm lesion **(C)** (4×, H&E) shows a discrete area of spongiosis and perivascular inflammation. **D,** (20×, H&E) Higher power shows prominent epidermal spongiosis with lymphocytic exocytosis and scattered intraepidermal eosinophils. *Black arrows* demonstrate infiltration with eosinophils both within the epidermis and throughout the dermis.

**Fig 3:**
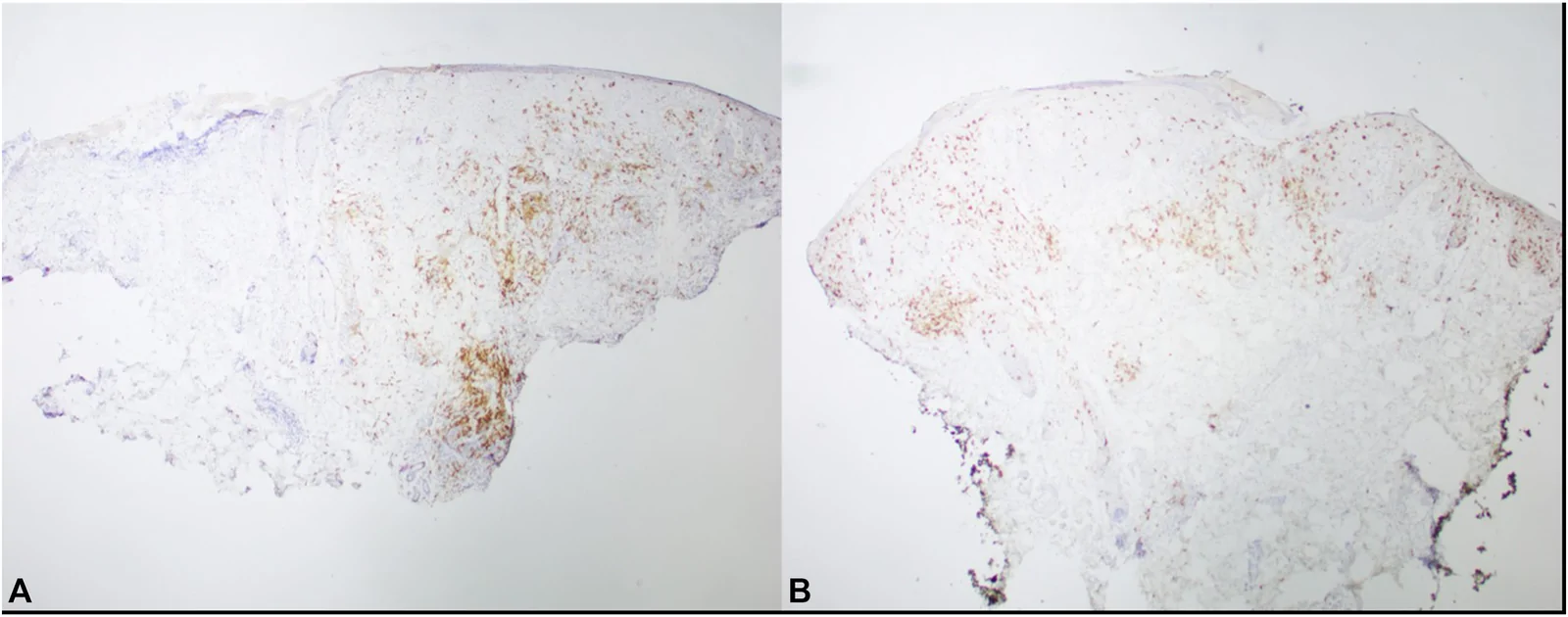
Case 1: Cd1α staining. Immunohistochemical staining demonstrating positive staining for Cd1α **(A)** back biopsy (4×) **(B)** arm biopsy (4×).

Punch biopsies from the chest and left arm were performed and showed dermal inflammatory infiltrate including eosinophils (Fig 2) and an increase in CD1α+ Langerhans cells, distributed as small clusters and nodules within the dermis (Fig 3). Following biopsy, the patient was started on topical tacrolimus and triamcinolone to help with pruritic symptoms. The histological differential diagnosis included EPF and early Langerhans cell histiocytosis. BRAF testing and bone marrow biopsy were performed and were both negative, favoring EPF. The patient was also diagnosed with eosinophilic esophagitis around this time, further supporting a potential overarching dysregulation of eosinophil mediated conditions. A second opinion was recommended at a large tertiary pediatric dermatology center, and it was agreed by this team that EPF was the most likely diagnosis.

As many cases of Infantile EPF are self-limiting, treatment focused on symptomatic alleviation. Tacrolimus and triamcinolone initially relieved itch. Treatment with 2.5 mg cetirizine twice daily was initiated, tacrolimus 0.03% was continued and mometasone 0.1% twice daily, 3 days a week, replaced triamcinolone. However, on follow up, the mometasone and triamcinolone proved ineffective, with the patient remaining highly pruritic with significant sleep disturbance. The patient was started on 200 mg injections of dupilumab every 4 weeks and responded well showing near complete response. Remaining small spots resolved within 24 hours after treatment with topical dapsone. He continues to be well-managed with dupilumab and topical dapsone, as needed, for breakthrough lesions.

### Case 2

A 25-year-old female patient was referred for evaluation of a recurrent rash along the forehead, scalp and behind the ears that had been ongoing since the age of 15. The rash had initially been thought to be eczema at an outside institution, and following biopsy, a working diagnosis of angiolymphoid hyperplasia with eosinophilia had been established. She had previously been unsuccessfully treated with topical steroids, topical calcineurin inhibitors and a trial of doxycycline, and was placed on dupilumab (biweekly, 300 mg/2 mL pen injector). She reported that flares occurred approximately every 3 weeks and lasted approximately 3 weeks. The lesions were described as itchy when inflamed with dark brown pigmentation remaining in previous areas of involvement. No other mucocutaneous or systematic symptoms were reported.

**Fig 4:**
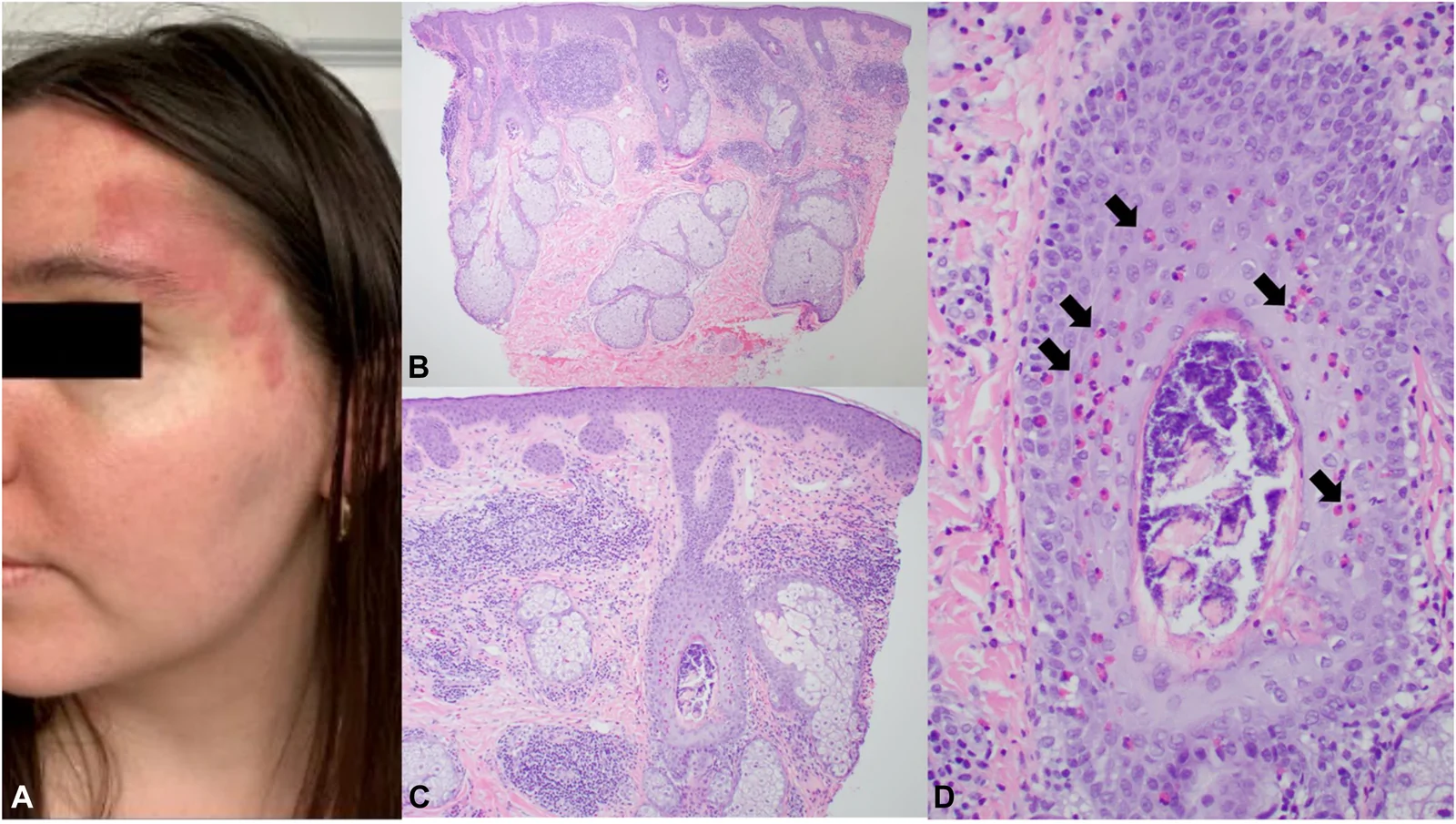
Case 2: Clinical presentation and histology. **A,** Erythematous, nonscaly, slightly raised papules and plaques on a background of hyperpigmentation on the left temple. **B,** (4× H&E) The punch biopsy from the temple demonstrates prominent perivascular and perifollicular inflammation. **C,** (10× H&E) Multiple follicles containing significant perivascular, intrafollicular, and perifollcular inflammatory cells including predominantly lymphocytes and eosinophils. Neutrophils are not readily appreciated and vessels are unremarkable. **D,** (20× H&E) *Black arrows* demonstrate prominent infiltration with eosinophils.

Clinical examination revealed erythematous, nonscaly, slightly raised papules and plaques on a background of hyperpigmentation on the bilateral temples predominately on the left forehead and temple with no scalp involvement (Fig 4). The histopathologic diagnosis from prior biopsy did not correlate with the clinical presentation, and another biopsy was recommended. The clinical differential included eosinophilic cellulitis, granuloma faciale, sarcoidosis, or connective tissue disorder with a less likely diagnosis of angiolymphoid hyperplasia with eosinophilia.

The punch biopsy findings revealed multiple follicles with intrafollicular eosinophils. Additionally, perivascular inflammation was seen without prominent vessels. Lymphocytes, histiocytes, occasional plasma cells, and increased numbers of eosinophils were noted with no perivascular neutrophils or perivascular fibrosis (Fig 4). The histology supported a diagnosis of eosinophilic folliculitis. Laboratory tests, including autoimmune workup, immunoglobulins, and eosinophils, were within expected ranges. HIV serology was negative.

After 6 months of treatment, the patient saw a reduction in severity and duration of flares but still was flaring monthly. She was then prescribed indomethacin (25 mg twice daily), which led to immediate improvement of the flair on her right temple. However, symptoms recurred, with significant pruritus on her left temple. The patient was then started on ruxolitinib, planning for follow-up in 2-3 months to assess treatment response.

## Discussion

Our first case was a 2-year-old male with eroded, crateriform papules and discrete follicular inflammation. The second was an immunocompetent female with a recurring rash, mimicking eczema, on the head and neck. Biopsies in both cases showed a significant number of intrafollicular, perifollicular, and perivascular eosinophils, with case 1 also demonstrating an increased CD1α positive cell population, atypical of EPF. These cases demonstrate how clinical, histologic and epidemiological characteristics can vary widely in EPF, and the importance of taking biopsy, physical characteristics and symptoms into account.

In our first case, dupilumab proved highly effective. This adds to a growing body of recent literature supporting dupilumab efficacy for EPF.^5^, ^6^, ^7^ In the second case, dupilumab provided only moderate improvement, with ruxolitinib further improving clearance. Compared to other cases successfully utilizing Janus kinase inhibitors for treatment, Janus kinase inhibition represented a second-line biologic.^8^, ^9^, ^10^ Given that EPF is often a diagnosis of exclusion, observation of indomethacin response can provide supportive evidence, with topical indomethacin being reported as effective in infantile EPF.^11^ For the first case, it is important to note that topical indomethacin was not evaluated and thus alternative diagnoses cannot be completely excluded.

EPF is a rare disease that can manifest variably both clinically and histopathologically, manifesting similarly to common dermatologic disorders, making diagnosis without biopsy difficult. We highlighted 2 different presentations EPF, both requiring close clinicopathologic correlation to arrive at the diagnosis. The cases were both managed effectively with biologics, suggesting promising options for refractory EPF cases.

### Declaration of generative AI and AI-assisted technologies in the writing process

None.

## Conflicts of interest

None disclosed.
